# Synergistic Effect of Multi-Walled Carbon Nanotubes and Graphene Nanoplatelets on the Monotonic and Fatigue Properties of Uncracked and Cracked Epoxy Composites

**DOI:** 10.3390/polym12091895

**Published:** 2020-08-23

**Authors:** Yi-Ming Jen, Jui-Cheng Huang, Kun-Yang Zheng

**Affiliations:** Department of Mechanical and Mechatronic Engineering, National Taiwan Ocean University No. 2, Pei-Ning Rd., Keelung 20224, Taiwan; DanteHuang@ntc.com.tw (J.-C.H.); best2944@hotmail.com (K.-Y.Z.)

**Keywords:** nanocomposite, multi-walled carbon nanotube (MWCNT), graphene nanoplatelet (GNP), synergistic effect, monotonic property, fatigue property, crack deflection effect

## Abstract

The fatigue properties of the polymer nanocomposites reinforced with a hybrid nano-filler system have seldom studied before. Accordingly, epoxy nanocomposites with various multi-walled carbon nanotube (MWCNT)/graphene nanoplatelet (GNP) filler ratios were prepared to study comprehensively the synergistic effect of the hybrid nano-fillers on the monotonic and cyclic mechanical properties of the nanocomposites. The quasi-statically tensile properties and fatigue-life curves were experimentally determined using uncracked bulk specimens. Additionally, pre-cracked specimens were utilized to study the fracture toughness and fatigue crack growth rate of the nanocomposites. A synergistic index based on the properties of the nanocomposites with individual types of filler was proposed to evaluate the synergistic effect of two employed nano-fillers on the studied properties. The index was verified to be a highly discriminatory tool to evaluate the synergistic effect of hybrid nano-fillers on the studied mechanical properties. The experimental results show that the composites with a MWCNT:GNP ratio of 1:9 have the higher monotonic and fatigue properties than those with other filler ratios. Adding appropriate amount of CNTs can prevent the agglomeration of GNPs. The flexible CNTs bridge adjacent GNPs to constitute a favorable network for load transfer. Moreover, there is a linear relationship between the static and fatigue strengths of the studied nanocomposites. Integrated analysis of experimental data and a fracture surface study reveals that the dispersion of nano-fillers influences the mechanical properties significantly. The crack deflection effect due to the path bifurcation caused by encountering the filler cluster and the filler bridging effect are the main reinforcement mechanism of the studied properties.

## 1. Introduction

With an increasing number of industrial applications, carbon particles with different nano-dimensionalities, such as fullerene, carbon black, nano-diamonds (NDs), carbon nanotubes (CNTs), graphite-family nano-sheets, graphene aerogels, etc.; have been widely employed to improve the mechanical, electrical, and thermal properties of polymer materials. Many synthesis efforts have been made to build effective functional groups on the surface of the nano-particles to improve adhesion with the polymer matrix. Moreover, various mixing techniques have been adopted to obtain uniform dispersion of nano-particles in the matrix because the degree of distribution of the reinforced particles influences the mechanical properties significantly. The mechanical properties of the polymers mixed with individual types of carbon nano-filler have been well studied, and some review works are available for references [1,2,3,4,5]. In general, adding a small amount of carbon nano-fillers can increase the mechanical properties of the polymers significantly, and the aggregates owing to the employment of excessive nano-fillers are deleterious to the mechanical properties. Theoretically, the reinforced achievements of carbon nano-particles on the mechanical properties of polymers increase with the dimensionality of the employed nano-reinforcements. However, the aggregation due to the larger aspect ratios of nano-fillers has detrimental influences on the mechanical properties of the nanocomposites.

Since each type of carbon nano-filler has unique geometrical characteristics, the synergistic effect of employing two types of carbon nano-filler as the reinforcements on the mechanical properties of the polymer materials has been studied in recent decades. Among various combinations of hybrid nano-fillers, CNTs and graphene-based nano-fillers are often considered as potential candidates to enhance the mechanical properties of neat polymers since the particular tube- and flake-shape features make the hybrid fillers have relatively larger aspect ratios than other combinations of carbon nano-fillers. Moreover, the filler ratio between the hybrid nano-fillers is another important issue to obtain the optimal mechanical properties of the polymer nanocomposites. In 2008, Li et al. studied the mechanical properties of epoxy nanocomposites reinforced with CNTs and graphene nanoplatelets (GNPs) [6]. No apparent synergistic effect of hybrid fillers was found on the flexural modulus and strength. However, the fracture toughness of the studied nanocomposites increases with the employed CNT contents when the total content of two fillers is kept constant at 2 wt %. Except for the quasi-static properties, the synergistic effect of GNPs and CNTs on the dynamic mechanical properties of the polyetherimide (PEI) composites was studied by Kumar et al. [7]. When the total filler loading is kept constant at 0.5 wt %, the dynamic storage moduli of the PEI nanocomposites with a filler ratio of 1:1 are higher than those with the individual type of nano-filler.

The synergistic effect of GNPs and CNTs on the tensile properties of epoxy was also investigated by Yang et al. in 2011 [8]. The nanocomposites with a CNT:GNP ratio of 1:9 have higher tensile modulus, tensile strength, and elongation than the nanocomposites with other filler ratios. Adding slight amount of CNTs plays an important role in bridging the adjacent GNPs and prevents the aggregation of GNPs. In the same year, the tensile properties of ultrahigh-molecular-weight polyethylene (UHMWPE) reinforced with graphene sheets (GNSs) and CNTs were experimentally studied by Ren et al. [9]. When the total content of the hybrid fillers is 0.5 wt % and the filler ratio of GNS:CNT is 1:3, the nanocomposites display higher tensile strength and modulus than those with other filler contents and filler ratios.

The influence of nano-particle size on the mechanical properties of polymers was investigated by Chatterjee et al. in 2012 [10]. They found that the epoxy-based nanocomposites with larger GNP flake size have higher mechanical properties than those with smaller GNP flake size. Furthermore, the hybrid filler nanocomposites with a CNT:GNP ratio of 9:1 have higher fracture toughness and flexural strength than those with other filler ratios. The synergistic effect of GNP/CNT and expanded graphite (EG)/CNT hybrids on the mechanical properties of styrene butadiene rubber (S-SBR) nanocomposites was studied by Das et al. [11]. It was reported that for either the S-SBR nanocomposites containing 15 phr (parts per hundreds) GNPs or those with 20 phr EGs, the quasi-static modulus and dynamic storage modulus increased with the amounts of CNTs. 

Li et al. proposed an innovative hybrid nano-filler by growing the CNTs on the GNPs in 2013 [12]. The tensile properties of the epoxy resins with the new type of hybrid fillers were examined and compared with those of traditional CNT/GNP/epoxy trinary nanocomposites. The results show that the tensile modulus, tensile strength, and fracture strain of the epoxy specimens with the hybrid nano-fillers are higher than the binary and trinary nanocomposites. The hybrid CNT-GNP fillers demonstrate good dispersion ability in the matrix and adequate interfacial strength between the nano-fillers and matrix. Zhang et al. employed functionalized graphene and CNTs to enhance the mechanical properties of poly(ether sulfone) (PES) composites [13]. When the mixed loading is 5 wt %, the PES composites with a graphene:CNT weight ratio of 1:1 show higher tensile modulus than those with other filler ratios. However, the tensile strength of the studied composites with hybrid fillers increases with the employed CNT loadings.

In 2014, Pradhan and Srivastava employed graphene and CNTs to enhance the tensile properties of silicone rubber using a simple solution mixing method [14]. The nanocomposites with two types of nano-fillers were found to have higher tensile strength and modulus than those with individual reinforcements. In the following year, Montes et al. studied the tensile properties of poly(vinyl alcohol) (PVA) based nanocomposites reinforced by cellulose nanocrystal-stabilized graphene (GR-CNC) [15]. The reinforcement effect of GR-CNC on the properties of PVA nanocomposites was also compared with graphene stabilized by an organic surfactant (GR-T) and CNC. The results show that PVA specimens with GR-CNC have higher tensile strength and modulus than those with individual type of nano-filler. Li et al. studied the synergistic effect of graphene and montmorillonite (MMT) clay on the tensile behavior of PVA [16]. It was reported that the nanocomposites with a graphene:MMT ratio of 3:1 displayed significant improvement in tensile strength and modulus when compared with the those of neat PVA. The magnitudes of the studied properties are even larger than the sum of those of the nanocomposites with an individual type of nano-filler. Besides, Cui et al. examined the synergistic effect of boron nitride (BN) and GNS on the mechanical properties of the polystyrene (PS) and polyamide 6 (PI) nanocomposites [17]. The results show that adding 1.5 wt % BN in the matrix of PS and PI nanocomposites containing 20 wt % GNSs can increase the tensile strengths and moduli of the binary nanocomposites markedly.

In 2015, Al-Saleh found that under fixed total contents of GNPs and CNTs, both the tensile strength and toughness of polypropylene increased with the CNT volume fraction employed in the preparation of nanocomposite specimens [18]. The bridging effect of CNTs between the GNP flakes was reported to be the main reinforcement mechanism. The following year, Moosa et al. studied the tensile properties of epoxy reinforced by CNTs and GNPs with a fixed filler ratio of 1:1 [19]. The tensile modulus was found to increase with the total contents of two employed nano-fillers. Furthermore, Wang et al. employed GNPs to enhance the tensile properties and the fracture toughness of the epoxy resin with 10 wt % carboxyl terminated butadiene acrylonitrile (CTBN) [20]. Two kinds of GNP with different diameters were added in the matrix to investigate the size effect of GNPs on the studied properties. The results reveal that the GNPs with larger diameter have higher reinforcement performance than those with smaller diameter. Adding 3 wt % GNPs in the CTBN/epoxy matrix displays significant improvement in fracture toughness when compared with the neat epoxy. Crack deflection, layer breakage and delamination of GNP layers are the mechanisms to improve the fracture toughness.

Ghaleb et al. added 0.5 vol.% graphene nano-powder and CNTs to study the synergistic effect of hybrid fillers on the tensile properties of epoxy based nanocomposites in 2017 [21]. The nanocomposites with a graphene:CNT ratio of 1:4 have the higher tensile strength and tensile modulus than the ones with other filler ratios. In the same year, graphene oxides (GO), CNT oxides (CO), and cross-linked GO-CO (LGC) were prepared by Wang et al. to compare the reinforcement effects on the tensile properties and toughness of the PI nanocomposites with GO/CNT, GO/CO, and LGC filler systems [22]. Experimental results show that the PI nanocomposites with LGC reinforcements have higher tensile strength and those with GO/CO fillers have higher toughness and elongations than other candidates. The amide bonds obtained by the proposed synthesis process provide strong interfacial between the nano-fillers and the PI matrix, and further improve the mechanical properties.

In 2018, Sahu et al. employed NDs, CNTs, and GNPs to study the synergistic effect of carbon nano-fillers with various dimensionalities on the mechanical properties of high-density polyethylene (HDPE) [23]. The nanocomposites with GNP/ND and CNT/GNP filler systems display higher stiffness/hardness and Young’s modulus than those with other hybrid filler systems, respectively. Riberio et al. studied the mechanical properties of the epoxy-based nanocomposites reinforced by hexagonal boron nitride (h-Br), GO and combined GO/h-Br filler systems [24]. The epoxy specimens with hybrid filler system show higher tensile strength and modulus than the ones with individual type of reinforcement when the contents of total fillers are kept constant at 0.5 wt %. Moreover, Min et al. employed the GOs and CNTs to enhance the tensile properties of PI [25]. The optimal GO:CNT ratio of 3:1 and 1:9 were obtained for the tensile strength and tensile modulus, respectively.

Recently, Shukla and Sharma employed amine functionalized multilayer graphene and CNTs as the reinforcements to study the synergistic effect of these two nano-fillers on the mechanical properties of epoxy nanocomposites [26]. The fillers with a graphene:CNT ratio of 1:3 display the highest synergistic effect on the tensile and flexural strengths and moduli of the epoxy when compared with the ones with other filler ratios. Wang et al. employed GOs and CNTs to improve the mechanical properties of shape memory epoxy [27]. The weight ratio between GOs and CNTs was kept at 1:1 in the preparation of the hybrid nanocomposites specimens. The experimental results show that the tensile strengths and the storage moduli of the hybrid nanocomposites are higher than those of the composites with individual type of nano-filler. 

Surveying past studies concerning the mechanical properties of the nanocomposites with nano-hybrid fillers reveals that the studied properties are focused on the quasi-static tensile/flexure properties and fracture toughness. Studies regarding the fatigue behavior of the polymer nanocomposites with carbon nano-filler reinforcements are relatively rare compared with the monotonic studies. The CNTs and carbon nanofibers (CNFs) are the most frequently used carbon nano-particles to enhance the fatigue behavior of the nanocomposites [28,29,30,31,32]. Furthermore, the effects of adding fullerene and graphene nano-sheets in the matrix to improve the fatigue strength of polymer nanocomposites have been reported in [33] and [34], respectively. In general, more remarkable improvement in the fatigue strength was observed than the quasi-static strength when the carbon nano-fillers were added in the polymer matrix. Moreover, Ladani et al. [35] found that the epoxy nanocomposites with one-dimensional reinforcements (CNFs) have lower crack propagation rates than those with two-dimensional nano-fillers (GNPs). The synergistic effect of hybrid carbon nano-fillers on the cyclic properties of the polymer nanocomposites was rarely studied. In 2011, Ismail et al. adding various contents of CNTs, i.e., 0, 0.5, 1, 3, and 5 phr, in the carbon black (CB)/natural rubber nanocomposites to study the synergistic effects of two carbon particles on the fatigue properties of the rubber composites [36]. The total content of employed CB and CNTs was kept constant at 30 phr. The experimental results reveals that the nanocomposites with 29.5 phr CB and 0.5 phr CNTs have the highest fatigue life compared with the composites with other filler ratios. Furthermore, the fatigue behavior of the similar composites reinforced by CB and CNT bundles (CNTBs) was studied by Dong et al. in 2015 [37]. Several types of specimens were prepared by replacing various amounts of CB with CNTBs to study the fatigue crack growth rates of the CB/natural rubber nanocomposites. The lowest fatigue crack growth rate was observed for the nanocomposites with 3 phr CNTBs and 16 phr CB. Shokrieh et al. [38] found that the flexural fatigue life of epoxy composites with 0.25 wt % graphene and 0.25 wt % CNFs are higher than those with individual type of reinforcement. 

Owing to their wide application, the mechanical properties of hybrid polymer nanocomposites have attracted much attention recently. Furthermore, the engineering components are frequently subjected to the fluctuating loading, the knowledge of the fatigue property of the novel material is important in the design and application stages. Accordingly, the purpose of this work is to study the synergistic effect of multi-walled carbon nanotubes (MWCNTs) and GNPs on the monotonic and cyclic mechanical properties of the uncracked and cracked epoxy nanocomposites. The weight ratio between two employed nano-fillers is the main considered variable to evaluate the synergistic effect of the hybrid nano-fillers on the studied properties of epoxy nanocomposites. The quasi-statically tensile properties and fatigue life characteristics are experimentally studied using the bulk specimens, and the mode I fracture toughness and fatigue crack propagation rates are investigated using the pre-cracked specimens. The fracture surfaces obtained after the tests were observed using a scanning electron microscope (SEM) to examine the reinforcement mechanism of hybrid nano-fillers on the studied mechanical properties.

## 2. Materials and Methods 

### 2.1. Materials and Preparation of Specimens

**Figure 1:**
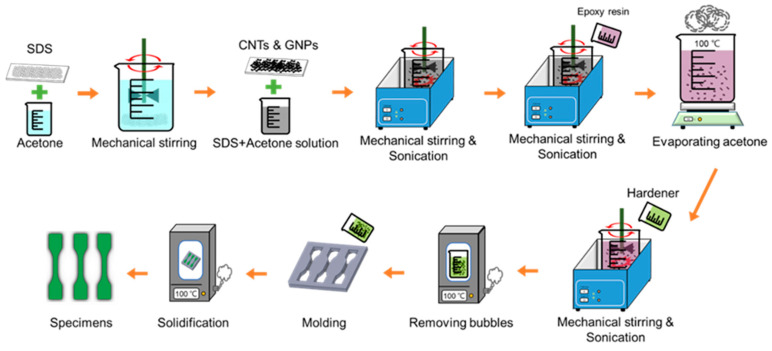
Fabrication process of the studied nanocomposite specimens.

The matrix of the studied nanocomposites was made of bisphenol A/F-based liquid epoxy resin and polyamine-based hardener. The epoxy system was supplied by Epotech Composite Corporation, Taiwan, with the designation of EPO-RT 90. To obtain low-medium viscosity, the resin was composed by 80% bisphenol A resin, 15% bisphenol F resin and 5 % modified epoxy resin. The polyamine based hardener was used to cure epoxy resin at room temperature. The mixture ratio between the employed resin and hardener was 100:35. The MWCNTs used were provided by Applied nanotechnologies Inc., US. The purity was larger than 95%. The employed MWCNT had a six-layered tubular structure. The diameter and the length of the MWCNTs ranged from 20–40 nm and 10–20 μm, respectively. The graphene nanoplatelets used were fabricated by Xiamen Knano Graphene Technology Co., China, with the designation of KNG-150. The purity of the employed GNP was larger than 99.5%, and specific surface area was about 40–60 mm^2^/g. The GNP was characterized with 10-layer graphene sheet structure. The diameter of the GNPs was approximately 4 μm, and the thickness was about 5 nm. To enhance the adhesion between the carbon nano-fillers and the epoxy matrix, sodium dodecyl sulfate (SDS) provided by Echo Chemical Co., Taiwan, was utilized as the surfactant. The structure of SDS has both hydrophilic and hydrophobic ends. It is often used to obtain homogeneous dispersion of carbon nano-fillers. The hydrophobic end sticks to the carbon filler, and the hydrophilic end improves the dispersion of the carbon fillers in the polymer matrix. Figure 1 shows the fabrication process of the studied nanocomposite specimens. The SDS was mixed with acetone with mechanical stirring for 30 min, then the required nano-fillers were added in the SDS solution with mechanical stirring and sonication for 10 min. Next, the epoxy monomer was mixed with the solution at room temperature with mechanical stirring for 10 min and sonication for 10 min. Subsequently the mixture was heated at 100 °C till the acetone had evaporated completely. After cooling to the room temperature, the hardener was added in the mixture with mechanical stirring for 10 min and sonication for 30 min. The mixture was cured in a vacuum oven for 1 h to remove the bubbles. The de-gassed mixture was poured into the molds with required shape and dimensions, and then cured in a vacuum oven at 100 °C continuously for 8 h to obtain the solidified specimens. The total contents of the two employed nano-fillers were set to be 0.2 and 0.4 wt % in the preparation of the nanocomposite specimens with hybrid fillers. These two magnitudes of total contents were determined because the optimal loadings for the tensile strengths of the MWCNT/epoxy and GNP/epoxy composites obtained in a preliminary study were 0.2 and 0.4 wt %, respectively. Moreover, the specimens with seven MWCNT:GNP filler ratios, i.e., 0:10, 1:9, 3:7, 5:5, 7:3, 9:1, and 10:0, were prepared to investigate the effect of filler ratio on the studied mechanical properties. Note that the specimens with the MWCNT:GNP ratios of 0:10 and 10:0 represented the specimens with only GNPs and MWCNTs, respectively. The neat epoxy specimens were also prepared for referential purpose. 

### 2.2. Tests of Mechanical Properties 

**Figure 2:**
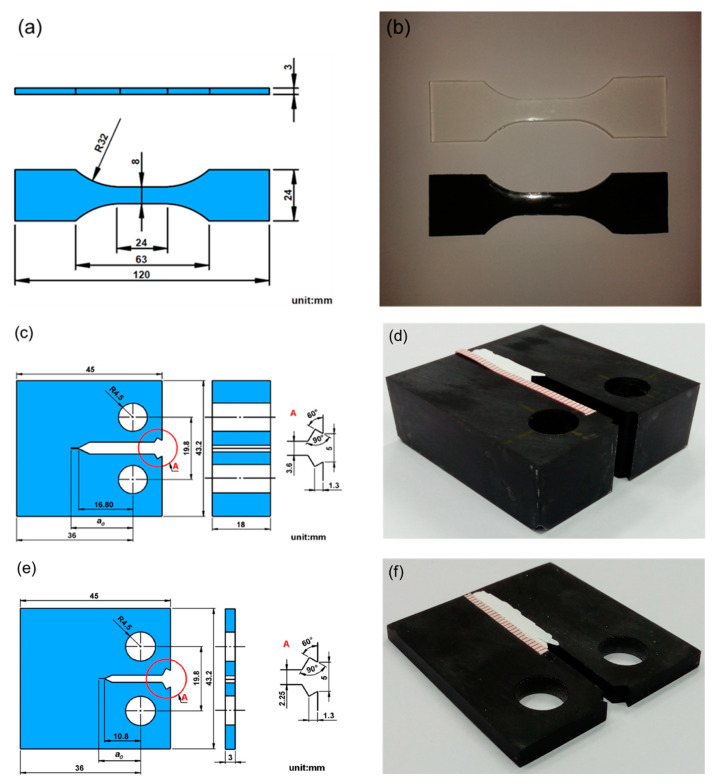
(**a**) Shape and dimensions of the tensile/fatigue specimen; (**b**) photograph of tensile/fatigue specimens; (**c**) shape and dimensions of the compact (CT) specimen employed in the fracture toughness tests; (**d**) photograph of the CT specimen employed in the fracture toughness tests; (**e**) shape and dimensions of the CT specimen employed in the crack propagation tests; and (**f**) photography of the CT specimen employed in the crack propagation tests.

Four types of tests, i.e., quasi-statically tensile tests, tension-tension fatigue tests, mode I fracture toughness tests, and fatigue crack propagation rate test, were performed to obtain the studied mechanical properties. All the tests were performed using an MTS 810 servo-hydraulic material testing system (MTS Systems Corporation; Eden Prairie, MN, USA). The shape and dimensions of the specimens for the monotonic tensile tests and the tension-tension fatigue tests were prepared according to the ASTM (American Society for Testing and Materials) standard D638 [39]. Figure 2a,b show the shape/dimensions and the photography of the tensile/fatigue specimens, respectively. The monotonic tensile tests were stroke controlled with the speed of crossheads of 0.01 mm/sec. An extensometer with 20 mm gage length was used to measure the strain. The tension-tension fatigue tests were load controlled. The stress ratio, defined as the ratio of minimum stress to maximum stress in one cycle, was set to be 0.1. The waveform of the cyclic loading is sinusoidal and the frequency is 5 Hz. The fatigue life *N_f_* is defined as the number of cycles corresponding to the specimen separation. 

The mode I fracture toughness of the studied nanocomposites was experimentally determined according to the ASTM standard D5045 [40]. The compact (CT) specimen shown in Figure 2c,d was prepared to perform the plane-strain fracture toughness tests. The pre-crack was initiated from the notch root by sliding a razor blade with thickness of 0.25 mm. The white correction liquid was painted along the predicted crack path for the visual observation of the crack behavior. The CT specimen was monotonically loaded with the crosshead speed of 0.01 mm/s and the mode I fracture toughness *K*_Ic_ can be obtained using the following equation [40]:

(1) $$K_{\mathrm{Ic}}=\left(\frac{P_{c}}{BW^{1/2}}\right)f\left(x\right)$$

where

(2) $$f\left(x\right)=\frac{\left(2+x\right)\left(0.886+4.64x-13.32x^{2}+14.72x^{3}-5.6x^{4}\right)}{{\left(1-x\right)}^{\frac{3}{2}}},\;x=\frac{a_{0}}{W}$$

In Equations (1) and (2), *B* and *W* are the thickness and the width of the specimens, respectively; *a*_0_ is the original crack length; *P_c_* is the critical applied load and can be determined according to the standard [40].

The fatigue crack propagation rate tests of the studied nanocomposites were conducted according to the ASTM standard E647 [41]. The shape/dimensions and photography of the CT specimen employed are shown in Figure 2e,f. The tests were performed under constant-amplitude load control with the load ratio (*P*_min_/*P*_max_) of 0.1. The waveform of the loading was sinusoidal and the frequency was 5 Hz. The crack opening displacement (COD) *v* was measured using a clip-on displacement gauge and the crack length *a* can be obtained using the following equations [41]:

(3) $$\frac{a}{W}=1.001-4.6695u_{x}+18.46u_{x}{}^{2}-236.82u_{x}{}^{3}+1214.9u_{x}{}^{4}-2143.6u_{x}{}^{5}$$

where

(4) $$u_{x}={\left\{\left[\frac{EvB}{P}\right]+1\right\}}^{\begin{array}{l} -1 \end{array}}$$

In Equation (4), *E* is the tensile modulus of the studied specimen. The aforementioned obtained crack length was also compared with the visual one observed using a travelling microscopy (Leica M80) to ensure the accuracy of crack length. Moreover, the crack intensity factor range Δ*K* can be expressed as:

(5) $$\Delta K=\frac{\Delta P}{B\sqrt{W}}\frac{\left(2+\frac{a}{W}\right)}{{\left(1-\frac{a}{W}\right)}^{\frac{3}{2}}}\left[0.886+4.64\frac{a}{W}-13.32{\left(\frac{a}{W}\right)}^{2}+14.72{\left(\frac{a}{W}\right)}^{3}-5.6{\left(\frac{a}{W}\right)}^{4}\right]$$

where Δ*P* is the load range (= *P*_max_ − *P*_min_) during a cycle.

After the tests, the fracture surfaces of the studied specimens were observed using a field emission SEM (JSM-6330F, JEOL Ltd., Tokyo, Japan) to examine the morphological characteristics and to determine the reinforcement mechanism of the hybrid filler system on the mechanical properties studied.

## 3. Results and Discussion

### 3.1. Monotonic Tensile Tests

**Figure 3:**
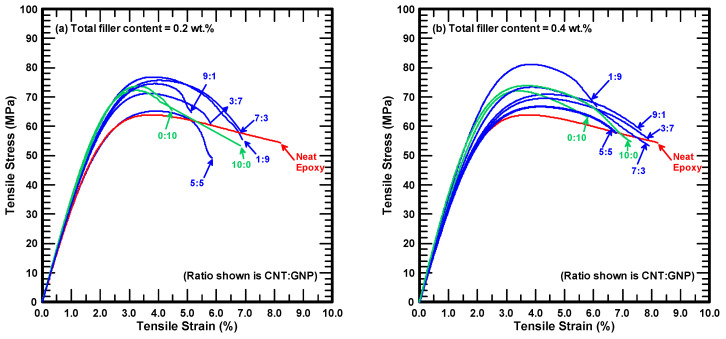
Stress-strain curves for the studied nanocomposites with total filler contents of (**a**) 0.2 wt %, (**b**) 0.4 wt %.

Figure 3a,b show the monotonic stress-strain curves of the studied nanocomposites with 0.2 and 0.4 wt % reinforcements, respectively. The plotted curves shown are selected as those whose ultimate strengths are closest to the average values obtained from three identical tests. The stress-strain curve for the pristine epoxy is also plotted in the two figures for comparative purpose. Similar trends of stress-strain curves for all nanocomposite specimens with various filler ratios are observed. These curves displayed the linearly elastic characteristics at the beginning stage of the tests, and were followed by the non-linear behavior till the peaks were attained. The ductile feature of the stress-strain curve was obvious until the specimen fractured.

**Figure 4:**
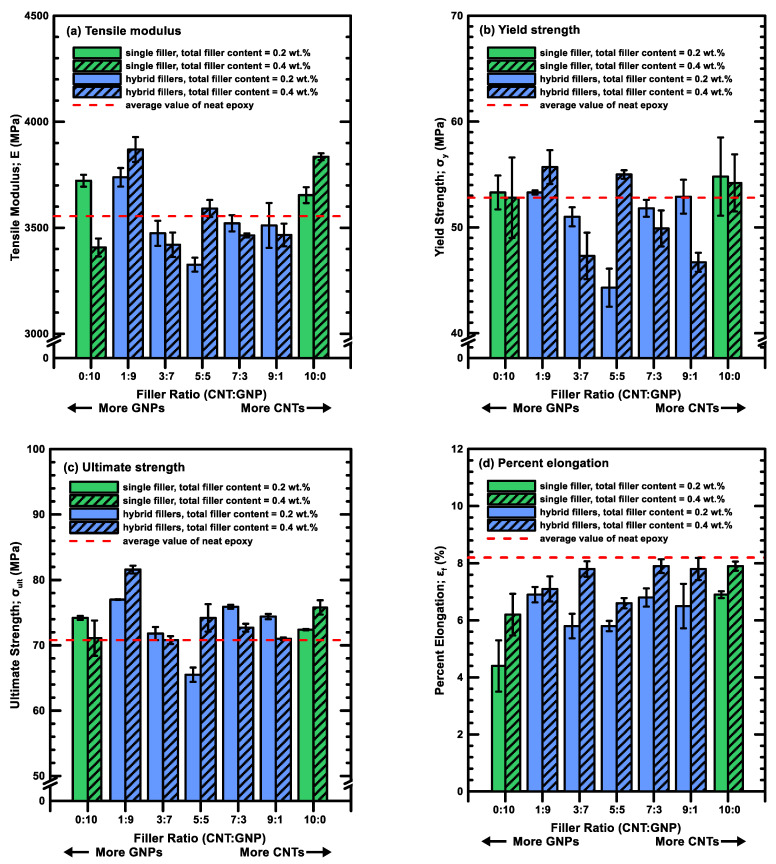
Monotonic properties for the nanocomposites with various filler ratios: (**a**) tensile modulus *E*, (**b**) yield strength *σ_y_*, (**c**) ultimate strength *σ_ult_*, and (**d**) percent elongation *ε_f_*.

Table 1 lists the experimental results of the monotonic properties obtained from the quasi-statically tensile tests, and Figure 4a–d show the variations of the tensile moduli *E*, yield strengths $\sigma_{y}$, ultimate strengths $\sigma_{ult}$, and percent elongations $\varepsilon_{f}$ of the hybrid nanocomposites with various filler ratios, respectively. Here the yield strength was obtained using the 0.2% offset method. Figure 4 displays that in general, adding one type of nano-filler in the matrix can slightly improve the tensile modulus, yield strength, and ultimate strength of the neat epoxy except for the composites with 0.4 wt % GNPs. Moreover, the aforementioned properties of the hybrid nanocomposites with appropriate filler ratios have higher improvements than those with an individual type of filler. Figure 4 shows that hybrid nanocomposites with a MWCNT:GNP ratio of 1:9 have higher tensile modulus and ultimate strength than those with other filler ratios. The hybrid nanocomposites with a total content of 0.4 wt % and a filler ratio of 1:9 increase the tensile modulus, yield strength, and ultimate strength of neat epoxy by 8.8%, 5.5% and 15.3%, respectively. By contrast, the ductility of the studied nanocomposites decreases significantly with the increase of the stiffness and strength. Figure 4d indicates that the percentage elongations of the studied nanocomposites are even lower than that of the neat epoxy, no matter single or dual types of nano-fillers are added in the epoxy matrix.

**Figure 5:**
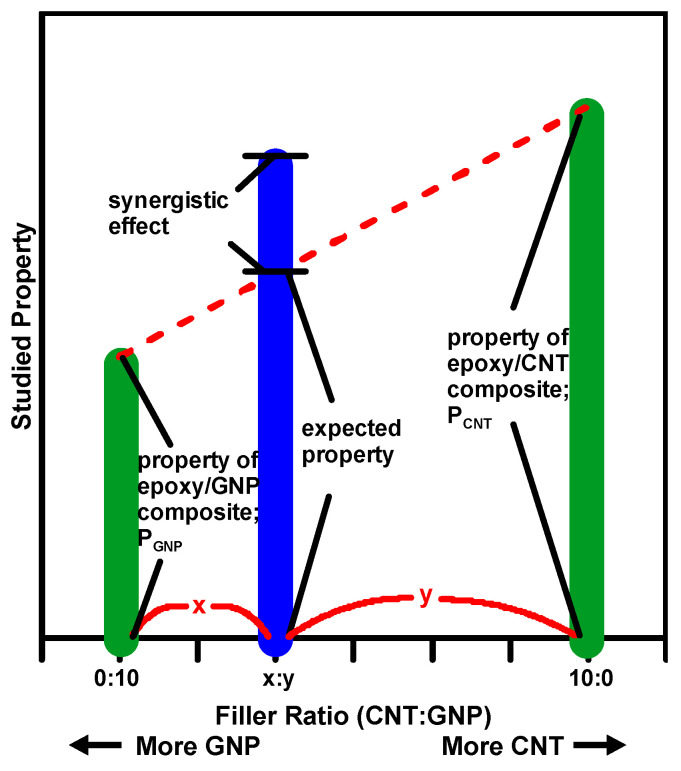
Conceptual schematic illustration for synergistic index.

In the study, a synergistic index χ is proposed to evaluate the synergistic effect of applying hybrid carbon nano-fillers in the epoxy matrix on the studied mechanical properties of the nanocomposites. Figure 5 shows the conceptual illustration of the proposed synergistic index. By contrast with the traditional rule of mixture where the properties of all constitutive components are considered, the expected property here is deduced based on the properties of the composites with individual type of filler and calculated according to the weight ratio of fillers. The difference between the experimental data and the expected value is used to evaluate the synergistic effect. That is, for the nanocomposites with a MWCNT:GNP ratio of x:y (x + y = 10), the synergistic index is expressed as:

(6) $$\chi \;\left(\%\right)=\frac{P_{hybrid}-\left(\frac{P_{CNT}x+P_{GNP}y}{10}\right)}{\frac{P_{CNT}x+P_{GNP}y}{10}}\times 100$$

where *P_hybrid_*, *P_CNT_* and *P_GNP_* represents the magnitudes of the studied properties for the nanocomposites with hybrid fillers, MWCNTs only (MWCNT:GNP = 10:0), and GNPs only (MWCNT:GNP = 0:10), respectively.

**Figure 6:**
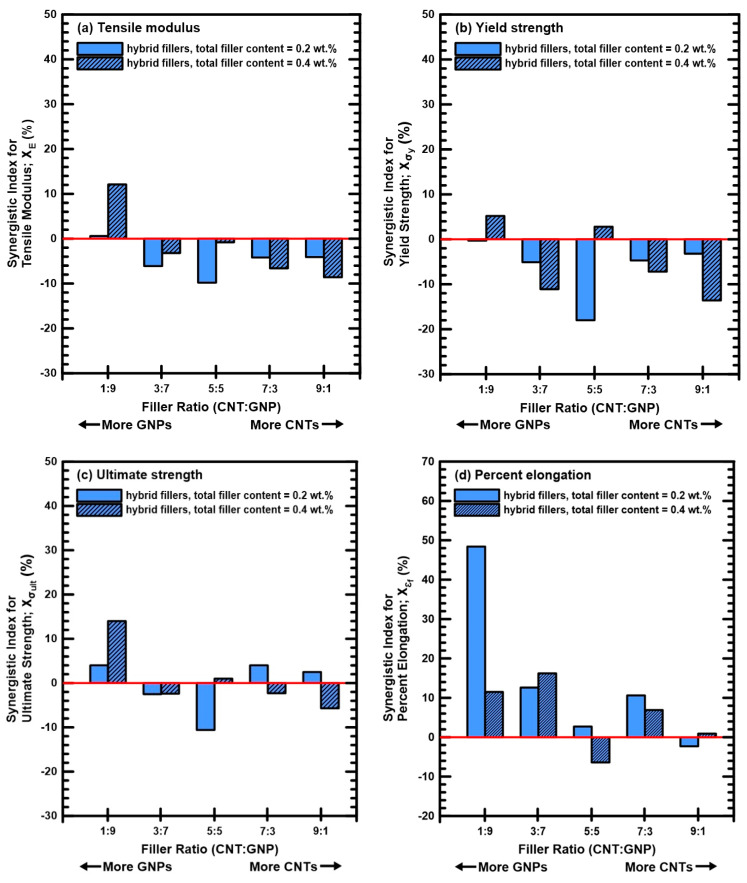
Synergistic indexes *χ* for the mechanical properties obtained in the monotonic tensile tests for the nanocomposites with various filler ratios: (**a**) tensile modulus *E*, (**b**) yield strength *σ_y_*, (**c**) ultimate strength *σ_ult_*, and (**d**) percent elongation *ε_f_*.

Table 2 lists the synergistic indexes for the studied monotonic properties of the nanocomposites with various filler ratios. Figure 6 shows the variation of the synergistic indexes with the employed filler ratios. It is evident that the strong synergistic effect can be found only for the nanocomposites with a MWCNT:GNP ratio of 1:9. The studied nanocomposites with other filler ratios display low or negative synergistic indexes for the monotonic properties. The results shown in Figure 6 illustrate that the proposed indexes have high degree of discrimination for the synergistic effect of two employed nano-fillers on the studied properties.

The experimental data of the tensile moduli of the studied nanocomposites were compared with the predicted results obtained using the Halpin-Tsai model [12,42,43]. The Halpin-Tsai model predicts the tensile modulus of the composites with randomly oriented MWCNT/GNP fillers *E* can be expressed as:

(7) $$E=[\frac{3}{8}\frac{1+2\left(l_{CNT}/d_{CNT}\right)\eta_{L}V_{CNT}+\left(2d_{GNP}/3t_{GNP}\right)\varsigma_{L}V_{GNP}}{1-\eta_{L}V_{CNT}-\varsigma_{L}V_{GNP}}\;+\frac{5}{8}\frac{1+2\eta_{T}V_{CNT}+2\varsigma_{T}V_{CNT}}{1-\eta_{L}V_{CNT}-\varsigma_{L}V_{GNP}}]\times E_{M}$$

where

(8) $$\eta_{L}=\frac{\left(E_{CNT}/E_{M}\right)-1}{E_{CNT}/E_{M}+2\left(l_{CNT}/d_{CNT}\right)};\;\eta_{T}=\frac{\left(E_{CNT}/E_{M}\right)-1}{\left(E_{CNT}/E_{M}\right)+2}$$

(9) $$\varsigma_{L}=\frac{\left(E_{GNP}/E_{M}\right)-1}{E_{GNP}/E_{M}+\left(2d_{GNP}/3t_{GNP}\right)};\;\varsigma_{T}=\frac{\left(E_{GNP}/E_{M}\right)-1}{\left(E_{GNP}/E_{M}\right)+2}$$

In Equations (7)–(9), *l_CNT_* and *d_CNT_* represent the average length and outer diameter of MWCNTs, respectively; *d_GNP_* and *t_GNP_* are the average diameter and thickness of GNPs, respectively; *V_CNT_* and *V_GNP_* are the volume fractions of MWCNTs and GNPs, respectively; *E*, *E_CNT_*, *E_GNP_*, and *E_M_* are the tensile moduli of the nanocomposites, MWCNTs, GNPs and epoxy matrix material, respectively. All the mechanical properties and geometric parameters used in Equations (7)–(9) are listed in Table 3. Since the nano-filler contents of the specimens were measured based on the weight unit system in the present study, the loadings of the employed nano-fillers with the volume unit system expressed in the Equations (7)–(9) can be obtained using the following equations:

(10) $$V_{CNT}=\frac{W_{CNT}}{W_{CNT}+W_{GNP}\left(\rho_{CNT}/\rho_{GNP}\right)+\left(1-W_{CNT}-W_{GNP}\right)\left(\rho_{CNT}/\rho_{M}\right)}$$

(11) $$V_{GNP}=\frac{W_{GNP}}{W_{CNT}\left(\rho_{GNP}/\rho_{CNT}\right)+W_{GNP}+\left(1-W_{CNT}-W_{GNP}\right)\left(\rho_{GNP}/\rho_{M}\right)}$$

where $\rho_{CNT}$ and *W_CNT_* are the density and weight fractions of MWCNTs, respectively; $\rho_{GNP}$ and *W_GNP_* are the density and weight fractions of GNPs; $\rho_{M}$ is the density of the matrix material.

**Figure 7:**
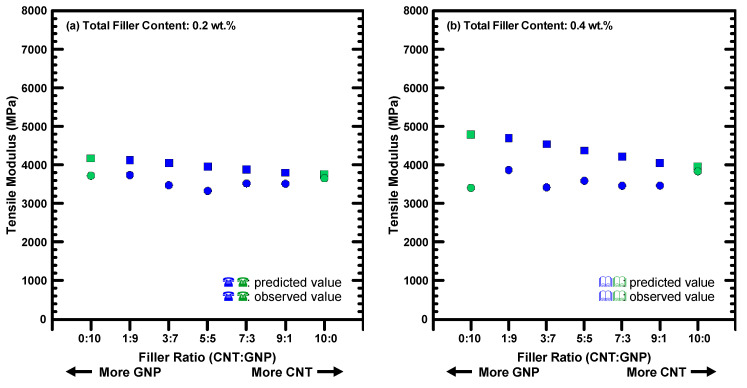
Comparison between the observed data and the predicted results for the tensile moduli of the nanocomposites with different filler ratios.

Figure 7 shows the comparison between the predicted results and experimental data of the tensile moduli for the hybrid nanocomposites studied with various filler ratios. The predicted moduli increase with the weight fractions of GNPs employed in the composites because the GNPs have larger aspect ratio than the MWCNTs. Moreover, all the predicted results are higher than the experimental values. The prediction error ranged from 2.9% to 19.2% and from 3.4% to 40.6% for the nanocomposites with 0.2 wt % and 0.4 wt % nano-fillers, respectively. Since the Halpin-Tsai model assumes that the orientations of the applied reinforcements are random, the degree of uniform dispersion of the nano-fillers influences the prediction accuracy significantly. The large prediction results of tensile moduli imply that the obtained mechanical properties are poor. Furthermore, the prediction error of the studied composites with 0.4 wt % nano-fillers is larger than that of the composites with 0.2 wt % nano-fillers. The high contents of nano-fillers may constitute agglomerates in the matrix, violating the assumption of Halpin-Tsai’s model.

### 3.2. Tensile Fatigue Tests

**Figure 8:**
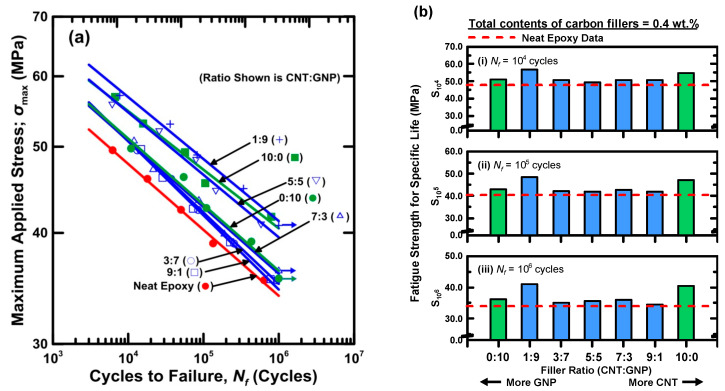
(**a**) S-N curves for the nanocomposites with various filler ratios, and (**b**) variations of fatigue strengths corresponding to various fatigue lives with filler ratios.

Since the ultimate strengths of studied nanocomposites with 0.4 wt % nano-fillers are higher than those with 0.2 wt % nano-fillers, the total content of hybrid nano-fillers employed in preparation of specimens for the subsequent tests is set as 0.4 wt %. Table 4 lists the experimental data obtained in the fatigue tests of the studied hybrid nanocomposites specimens with different filler ratios, and Figure 8a shows the stress-life (S-N) curves of the studied nanocomposites. The relationship between the applied maximum stress $\sigma_{\max}$ and the fatigue lives of the studied nanocomposites *N_f_* is described using a power-law equation:

(12) $$\sigma_{\max}=aN_{f}{}^{b}$$

where *a* and *b* are the fatigue strength coefficient and the fatigue strength exponent, respectively. As shown in Figure 8a, the pattern of a power law equation is a straight line in the log-log scale diagram, and the fitting results of the parameters *a* and *b* for all types of specimens are also listed in Table 4. The coefficients of determination R-squared for the fitting results of all S-N curves are higher than 0.97, indicating that the power law is appropriate to correlate the fatigue life with the applied maximum stress. The nanocomposites with individual type of filler increase the fatigue strength of neat epoxy markedly. Moreover, adding hybrid carbon fillers with appropriate filler ratios further improve the fatigue strength of the nanocomposites with individual type of filler. Figure 8b shows the fatigue strengths corresponding to 10^4^-, 10^5^-, and 10^6^-cycle fatigue lives (S_10_^4^, S_10_^5^, and S_10_^6^) for all types of studied specimens. Here the fatigue strength represents the applied maximum stress corresponding to a specific fatigue life. Figure 8b indicates that adding MWCNTs and GNPs individually in the epoxy approximately increases the fatigue strength of epoxy by 16 and 5%, respectively. However, adding hybrid nano-fillers with various filler ratios presents a different improvement effect on fatigue strength. All the hybrid nanocomposites with various filler ratios show higher fatigue strengths than the neat epoxy, however the fatigue resistance ability of most types of specimen is lower than that of the specimens with only MWCNTs. Only the nanocomposites with a MWCNT:GNP ratio of 1:9 show the highest fatigue strength among the studied nanocomposites with different filler ratios. It demonstrates that only mixing hybrid carbon nano-fillers with specific filler ratios in the matrix can display a conspicuous synergistic effect on the fatigue strength of polymer composites.

**Figure 9:**
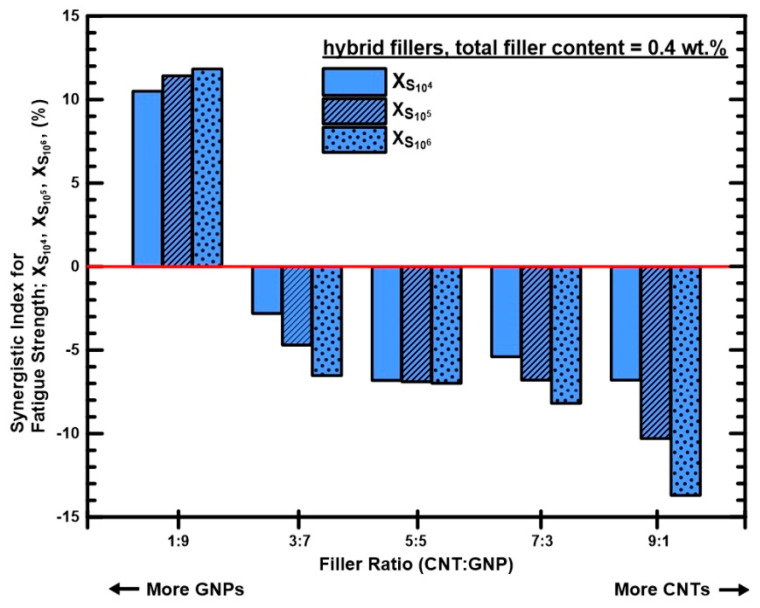
Variation of synergistic indexes *χ* of the fatigue strengths at 10^4^-, 10^5^-, and 10^6^-cycle lives of the nanocomposites with the employed filler ratios.

Figure 9 shows the variation of synergistic indexes for the fatigue strengths at low, medium, and high cycle ranges with employed filler ratios. The corresponding data are also listed in Table 2. Only the specimens with a MWCNT:GNP ratio of 1:9 display positive synergistic effect, and the collaborative effect of two nano-fillers increases with the fatigue lives. Moreover, it is evident that the proposed synergistic index plays as a high discrimination parameter to assess the co-working benefits of MWCNTs and GNPs on the improvement of fatigue strength.

**Figure 10:**
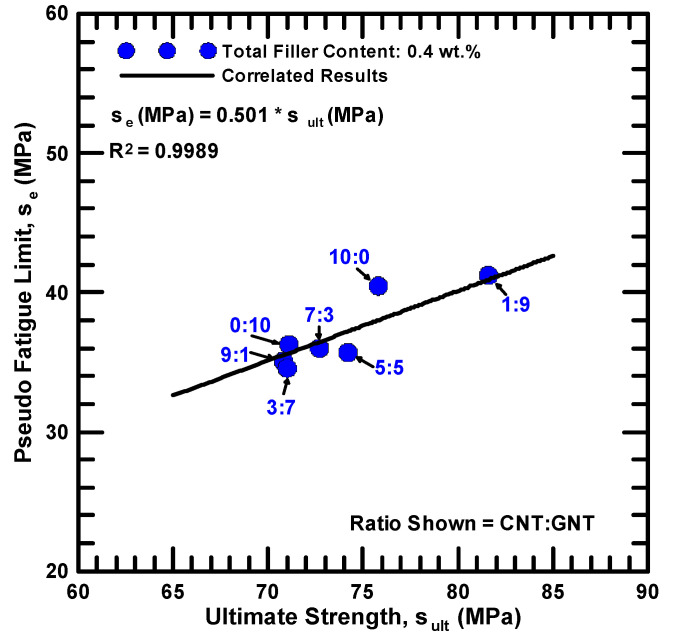
Relationship between the pseudo fatigue limits *σ_e_* and the ultimate strength *σ_ult_* for the studied nanocomposites with various filler ratios.

To study the correlation between the monotonic and cyclic strengths of the studied hybrid nanocomposites, Figure 10 depicts the relationship between the pseudo fatigue limit and the ultimate strength of the studied nanocomposites with various filler ratios. Here the pseudo fatigue limit is defined as the applied maximum stress corresponding to one million cycles (S_10_^6^). It shows that a linear relationship between the static and fatigue strengths can be observed. It demonstrates that no matter what filler ratio is designed and employed in the preparation of the nanocomposites, the fatigue limit can be predicted from the monotonic strength. Moreover, the slope of 0.5 for the fitting results is similar to the behavior of wrought steels [44].

### 3.3. Mode I Fracture Toughness Tests

**Figure 11:**
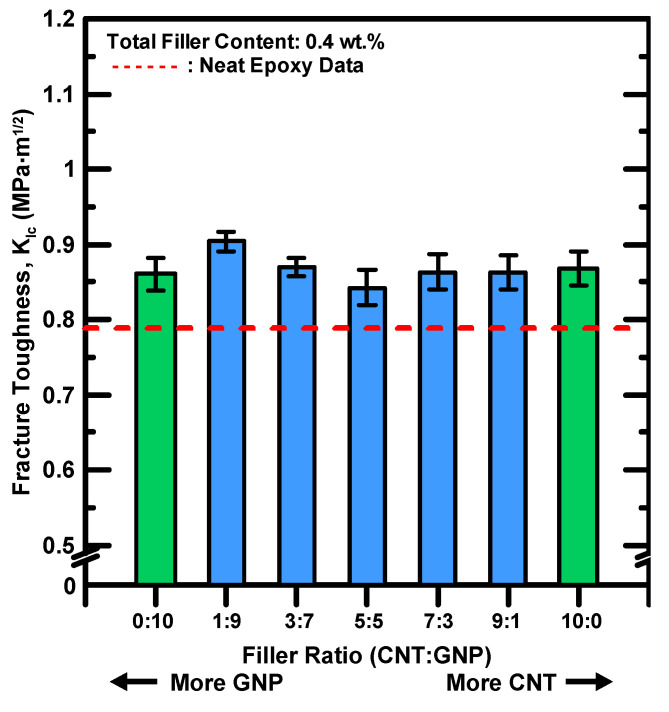
Variation of Mode I fracture toughness *K_IC_* of the studied nanocomposites with various filler ratios.

**Figure 12:**
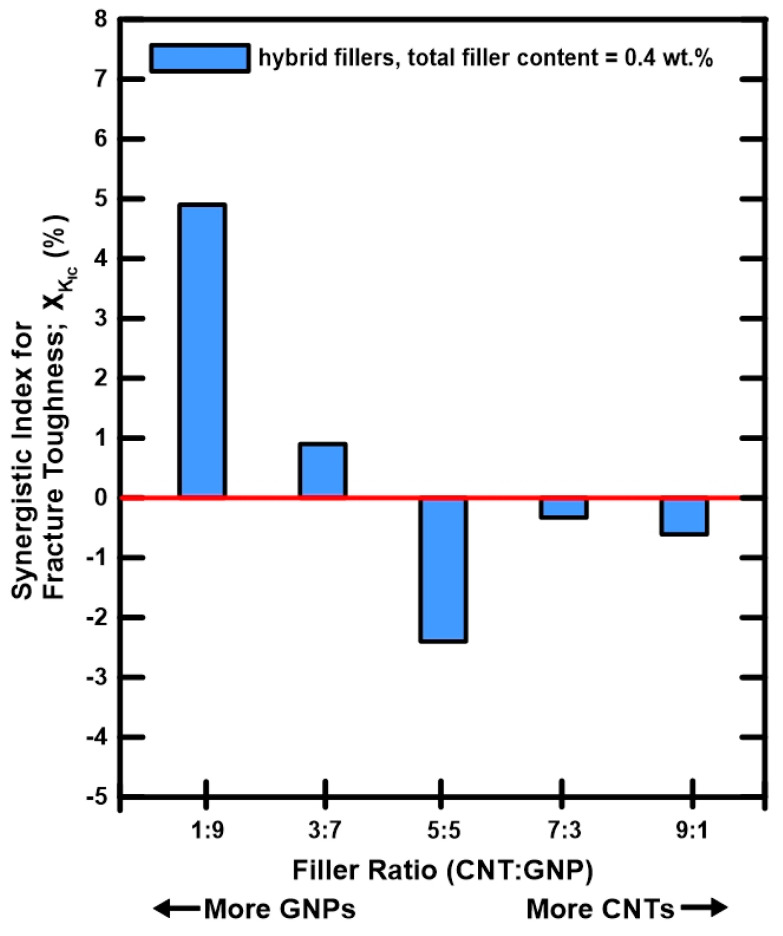
Synergistic indexes *χ* for the Mode I fracture toughness of the nanocomposites with various filler ratios.

Table 5 lists the experimental results of mode I fracture toughness for the studied nanocomposites with different filler ratios. The results are also shown in Figure 11, in which the composites with individual type of nano-filler are found to enhance the fracture toughness of neat epoxy evidently. The MWCNT/epoxy and GNP/epoxy nanocomposites increase the fracture toughness of the pristine epoxy by 10.1% and 9.2%, respectively. Moreover, adding hybrid nano-fillers displays similar improved effect on the fracture strength of the epoxy. Notable results are found for the hybrid nanocomposites with a MWCNT:GNP ratio of 1:9, which increase the fracture toughness of epoxy by 14.7%. The synergistic indexes for the mode I fracture toughness of the hybrid nanocomposites with various filler ratios are given in Table 2. Figure 12 presents the variation of the synergistic indexes for the fracture toughness with the employed MWCNT:GNP ratios of the hybrid nanocomposites. It is evident that the hybrid nanocomposites with a MWCNT:GNP ratio of 1:9 display a high synergistic effect on the studied fracture property. By contrast, the nanocomposites with other filler ratios show low or negative synergistic indexes. Once again the proposed synergistic indexes provide a contrastive tool to evaluate the synergistic effect of two nano-fillers on the improvement of fracture property.

### 3.4. Fatigue Crack Growth Rate Tests

**Figure 13:**
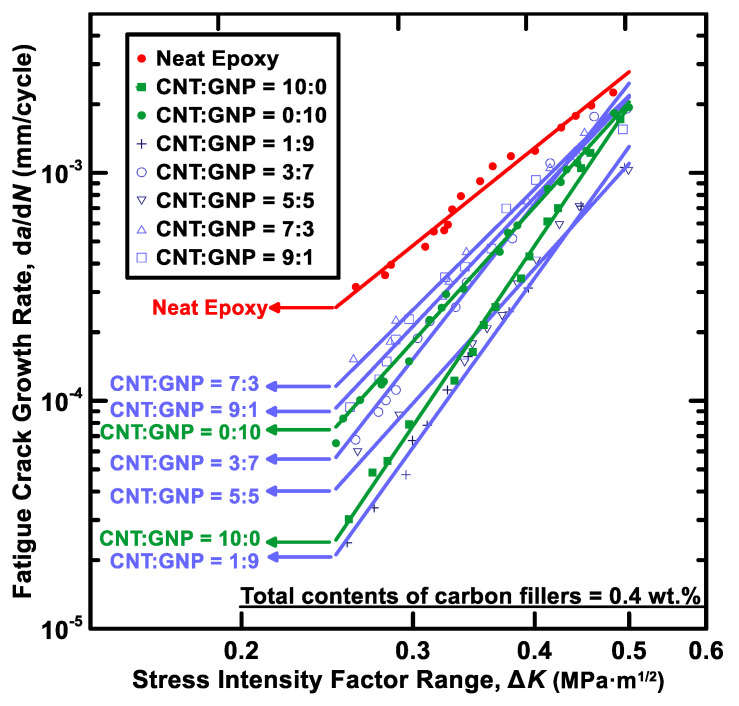
Relationship between the crack propagation rates *da/d*N and the stress intensity factor ranges Δ*K* for the studied nanocomposites with different filler ratios.

**Figure 14:**
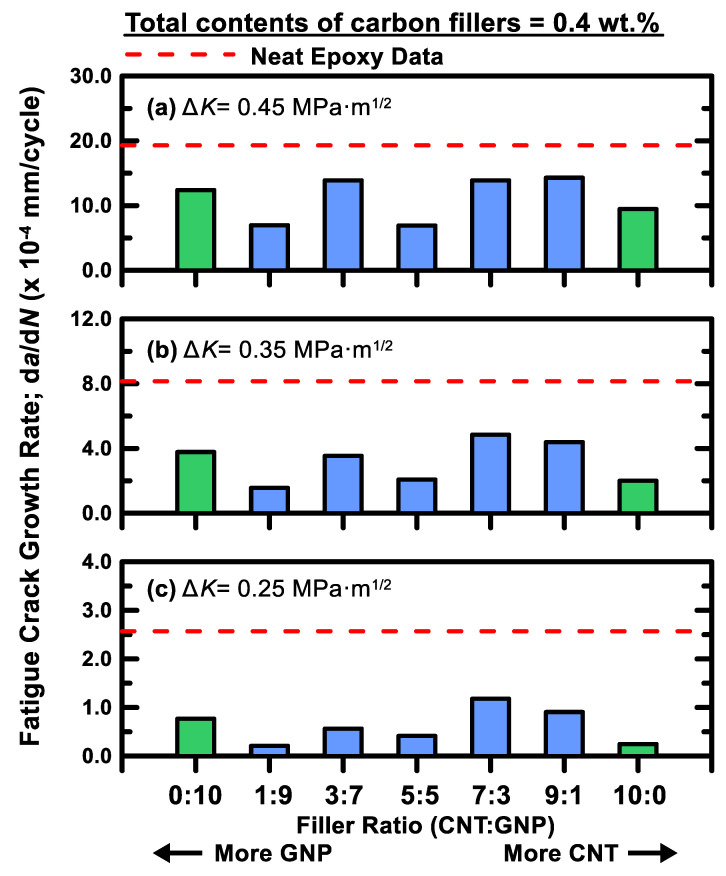
Comparison between the crack propagation rates of the nanocomposites with various filler ratios at Δ*K* = (**a**) 0.45; (**b**) 0.35; (**c**) 0.25 MPa·m^1/2^.

The fatigue crack propagation rates d*a*/d*N* of the studied hybrid nanocomposites with different filler ratio was experimentally studied using cracked specimens. The Paris law was used to describe the correlation between the fatigue crack propagation rates and the stress intensity factor ranges:

(13) $$\frac{da}{dN}=C{\left(\Delta K\right)}^{m}$$

where *C* and *m* are material constants. The fitting results of the material constants for the studied nanocomposites with various filler ratios are listed in Table 6. The coefficients of determination for the fitting results are higher than 0.94, indicating the employed power-law model is suitable to characterize the relationship between the crack growth rates and the stress intensity ranges. The experimental data and the fitting results using Paris law are shown in Figure 13. The power-law model was depicted as a straight line in the log-log diagram. Moreover, Figure 14a–c present the crack growth rates of all types of specimens at the stress intensity ranges of 0.45, 0.35, and 0.25 MPa, respectively. It indicates that adding a single type of nano-filler in the polymer matrix can reduce the crack propagation rate of neat epoxy significantly. Moreover, adding hybrid nano-fillers is also helpful for resisting the fatigue crack growth, and the stronger suppression effect on crack growth is observed at lower stress intensity rages. Whether the ability to inhibit the crack growth of hybrid filler system is higher than that of a single-filler system depends strongly on the filler ratios employed in the preparation of hybrid nanocomposites. The crack propagation rate of the composite specimens with a MWCNT:GNP ratio of 1:9 display the lowest crack growth rates among all the studied specimens with different filler ratios. The crack growth rate of the specimen with this specific filler ratio is 12 times lower than that of neat epoxy, and slightly lower than that of MWCNT/epoxy composites.

### 3.5. Facture Surfaces Study

**Figure 15:**
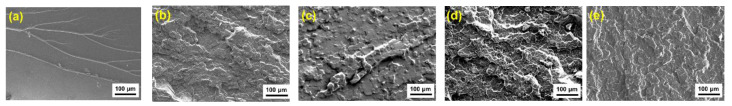
Scanning electron microscope (SEM) images of the fracture surfaces obtained after the monotonic tensile tests for (**a**) the specimens of neat epoxy and those with MWCNT:GNP ratios of (**b**) 10:0, (**c**) 0:10, (**d**) 3:7, and (**e**) 1:9.

Figure 15a–e are the low magnification SEM images (×200) of the fracture surfaces obtained after the monotonic tensile tests for specimen of neat epoxy and the ones with MWCNT:GNP ratios of 10:0, 0:10, 3:7, and 1:9, respectively. Comparing these characteristic fracture surfaces reveals that the neat epoxy has relatively smooth fracture surface (Figure 15a) than the composites with nano-fillers. The rough surfaces with obvious peak and valley-like feature are observed for the composites with individual type of nano-filler (Figure 15b,c). It implies that the nano-fillers block the development of fracture surfaces along the crack plane. The crack deflection effect makes the fracture surface rough and further improves the mechanical properties. Furthermore, the SEM images of composites with hybrid fillers (Figure 15d,e) present rougher surfaces than those with a single type of nano-filler. The denser peak and valley-like surfaces present that the synergistic effect of employed hybrid nano-fillers contributes a stronger crack deflection effect, and higher monotonic properties of the composites are obtained.

**Figure 16:**
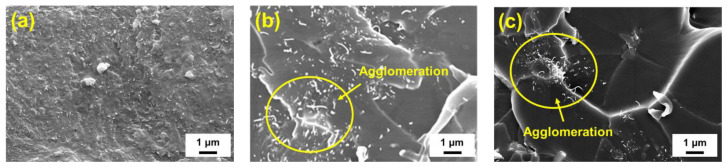
Enlarged SEM images of the quasi-static fracture surface for the studied nanocomposites with the MWCNT:GNP ratio of (**a**) 1:9, (**b**) 3:7 and (**c**) 9:1.

Figure 16a–c are the enlarged SEM images (×10,000) of the quasi-static fracture surface for the studied hybrid nanocomposites with the MWCNT:GNP ratio of 1:9, 3:7 and 9:1, respectively. Comparing the three SEM images reveals that the nano-fillers of the composites with a MWCNT:GNP ratio of 1:9 disperse more uniformly in the epoxy matrix than those of the other two nanocomposites (Figure 16a). Theoretically, the two-dimensional flake structure of GNPs provides larger contact area in the polymer matrix than the structure of one-dimensional carbon nano-fillers. This implies that the nano-fillers with higher dimensionality can improve the mechanical of polymers more efficiently. However, the π-π interaction and van der Waals force between the graphene layers make the GNPs to form aggregation easily. When optimal content of CNTs is added between the GNPs, the flexible CNTs can suppress the gathering of GNPs [21,45]. Furthermore, the tortuous CNTs form bridging and network between the GNPs, which are benefit to load transfer, further improve the mechanical properties. The similar reinforcing mechanism and the spatial configuration of CNTs and GNPs can be found in [8]. In Figure 16b,c, the clusters of nano-fillers are evident and the fractures are found to be initiated from these sites. Non-uniform distribution of nano-particles in the matrix has a detrimental effect on the static strength of polymer nanocomposites. The agglomeration of nano-fillers results in the stress concentrations and reduces the efficiency of load transfer. Moreover, the vicinity of the aggregates is likely to cause micro-voids or defects because the viscous polymer is not easy to fill the spaces between the nano-fillers of clusters during solidification. The low values of monotonic data of the aforementioned nanocomposites confirm the adverse influence of agglomeration on the mechanical properties.

**Figure 17:**
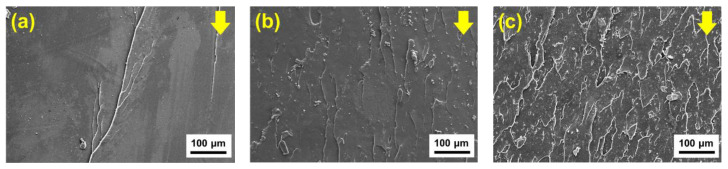
SEM images of the fatigue failure surfaces for the pre-cracked nanocomposites of (**a**) neat epoxy and the ones with MWCNT:GNP ratios of (**b**) 7:3 and (**c**) 1:9; the arrow marks indicate the direction of crack propagation.

**Figure 18:**
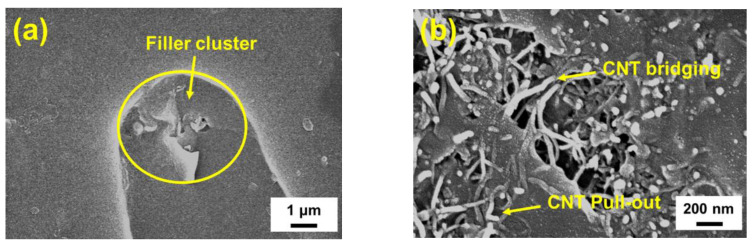
(**a**) Enlarged SEM images of crack bifurcation of the nanocomposites with a MWCNT:GNP ratio of 1:9. (**b**) SEM images of CNT bridging and pull-out of the nanocomposites with a MWCNT:GNP ratio of 9:1.

Figure 17a–c display the SEM images (×200) of the fatigue failure surfaces of the pre-cracked neat epoxy specimen and the ones with MWCNT:GNP ratios of 7:3 and 1:9 respectively. The arrow marks represent the directions of fatigue crack growth. The smooth fracture surface was observed for the neat epoxy specimens (Figure 17a), demonstrating that the fatigue crack propagated on the same plane and the brittle failure predominated the fracture characteristics. Figure 17b,c show that the rough fracture surfaces of the nanocomposites reinforced with hybrid nano-fillers. These fracture surfaces have features in the form of flow patterns. Furthermore, comparing the fatigue data with the corresponding fracture surfaces reveals that the nanocomposites with denser flow-pattern fracture surfaces have slower crack propagation rates. The morphological characteristics of fracture surfaces revealed that the cracks encountered the obstacles during growth. The crack was pinned and then bifurcated into upper and lower surfaces at different heights to bypass the obstacles. Figure 18a shows the enlarged SEM images (×10k) of crack bifurcation of the studied nanocomposites. The cluster of MWCNTs and GNPs obstructed the crack propagation. The crack bypassed the cluster by bifurcating into two surfaces and a narrow band was observed behind the filler cluster. The similar mechanism has been observed within graphene-based nanocomposites [20,46,47,48]. Since more energy dispassion is needed to bypass the nano-fillers by bifurcating the crack fronts, the ability to resist the fatigue failure is further improved. Additionally, for the nanocomposites with plenty of CNTs, the pull-out of CNT and CNT bridging are the main enhancement mechanisms of mechanical properties. The reinforcing effect by nanoparticle bridging and the push-pull mechanism has been also observed within the CNT-epoxy nanocomposites [49], GNP based epoxy nanocomposites [49,50], and nanocomposites with hybrid nano-fillers [51]. Figure 18b shows the SEM image (×5000) of the nanocomposite specimen with a MWCNT:GNP ratio of 9:1. Because more energy is needed to develop damage or fracture by pulling-out the CNTs from the matrix or breaking the CNT bridging across the matrix gap, the studied properties of the CNT-rich nanocomposites can be improved effectively.

## 4. Conclusions and Recommendations

The monotonic and fatigue properties of the MWCNT/GNP/epoxy nanocomposites were studied comprehensively by performing the quasi-statically tensile test, constant amplitude fatigue test, mode I fracture toughness test and fatigue crack growth rate test. A synergistic index was introduced to assess the synergistic effect of the hybrid filler system on the studied properties. Experimental results show that adding individual types of nano-filler in the matrix can improve the monotonic and fatigue properties of the neat epoxy. Moreover, the hybrid nanocomposites with appropriate filler ratios can further increase these mechanical properties. In the study, the nanocomposites with a MWCNT:GNP ratio of 1:9 present the optimal synergistic effect of the hybrid fillers on the studied properties. Adding a slight amount of CNTs in the matrix can prevent the agglomeration of GNPs and form a beneficial bridging network between the GNPs. Although diverse results of the optimal filler ratios on the mechanical properties of the trinary nanocomposites have been reported before, the fatigue strength was found to be proportional to the quasi-static strength regardless of the employed filler ratio. The fracture surface study reveals that the degree of uniform dispersion of nano-fillers and the crack deflection effect caused by the bifurcation of crack path affect the mechanical properties remarkably.

After understanding the synergistic effect of CNTs and GNPs on the monotonic and fatigue properties, the mechanical behavior of the nanocomposites with hybrid nano-fillers subjected to the transient loading, such as impact or collision, is another topic worth studying. In addition, since the properties of polymer materials are sensitive to temperature and humidity, knowledge of the hydrothermal effect on the mechanical properties of the studied nanocomposites with hybrid fillers is needed for the application of the novel nanocomposites in adverse environments.

## Figures and Tables

**Table 1:** Experiment results of monotonic mechanical properties for the nanocomposites with different filler ratios.

Filler Ratio	Monotonic Tensile Properties			
MWCNT:GNP	Tensile Modulus, *E* (MPa)	Yield Strength, *σ_y_* (MPa)	Ultimate Strength, *σ_ult_*(MPa)	Percent Elongation, *ε_f_* (%)
*Neat epoxy*	3554.7 ± 70.5	52.8 ± 2.7	70.8 ± 0.1	8.2 ± 0.54
*Total filler content = 0.2 wt %*				
0:10	3722.4 ± 28.1 (+4.7) ^1^	53.3 ± 1.6 (+0.9)	74.2 ± 0.3 (+4.8)	4.4 ± 0.89 (−46.3)
1:9	3738.2 ± 43.9 (+5.2)	53.3 ± 0.2 (+0.9)	77.0 ± 0.04 (+8.8)	6.9 ± 0.27 (−15.9)
3:7	3474.7 ± 59.2 (−2.2)	51.0 ± 0.9 (−3.41)	71.8 ± 1.0 (+1.4)	5.8 ± 0.43 (−29.3)
5:5	3326.1 ± 32.7 (−6.4)	44.3 ± 1.8 (−16.1)	65.5 ± 1.1 (−7.5)	5.8 ± 0.18 (−29.3)
7:3	3521.0 ± 38.2 (−0.9)	51.8 ± 0.8 (−1.9)	75.9 ± 0.3 (+7.2)	6.8 ± 0.32 (−17.1)
9:1	3511.4 ± 106.1 (−1.2)	52.9 ± 1.6 (+0.2)	74.4 ± 0.4 (+5.1)	6.5 ± 0.78 (−20.7)
10:0	3654.5 ± 37.6 (+2.8)	54.8 ± 3.7 (+3.8)	72.4 ± 0.1 (+2.3)	6.9 ± 0.12 (−15.9)
*Total filler content = 0.4 wt %*				
0:10	3407.2 ± 42.2 (−4.1)	52.8 ± 3.8 (+0/.0)	71.1 ± 2.7 (+0.4)	6.2 ± 0.73 (−24.4)
1:9	3868.7 ± 58.8 (+8.8)	55.7 ± 1.6 (+5.5)	81.6 ± 0.6 (+15.3)	7.1 ± 0.44 (−13.4)
3:7	3420.8 ± 57.6 (−3.8)	47.3 ± 2.2 (−10.4)	70.8 ± 0.6 (+0.0)	7.8 ± 0.27 (−4.9)
5:5	3590.6 ± 40.1 (+1.0)	55.0 ± 0.4 (+2.0)	74.2 ± 2.1 (+4.8)	6.6 ± 0.18 (−19.5)
7:3	3463.6 ± 9.7 (−2.6)	49.9 ± 1.7 (−5.5)	72.7 ± 0.6 (+2.7)	7.9 ± 0.24 (−3.7)
9:1	3465.7 ± 53.7 (−2.5)	46.7 ± 0.9 (−11.6)	71.0 ± 0.2 (+0.3)	7.8 ± 0.39 (−4.9)
10:0	3835.0 ± 16.1 (+7.9)	54.2 ± 2.7 (+2.7)	75.8 ± 1.1 (+7.1)	7.9 ± 0.16 (−3.7)

^1^ The value in the parentheses represents the percent improvement of the studied property compared with the data of neat epoxy.

**Table 2:** Synergistic indexes for the studied mechanical properties. (%).

	Filler Ratio	1:9	3:7	5:5	7:3	9:1
Studied						
Properties						
*Monotonic tensile properties* ^1^						
Tensile modulus		0.6/12.1	−6.1/−3.2	−9.8/−0.8	−4.2/−6.6	−4.1/−8.6
Yield strength		−0.3/5.2	−5.1/−11.1	−18.0/2.8	−4.7/−7.2	−3.2/−13.6
Ultimate strength		4.0/14.0	−2.5/−2.4	−10.6/1.0	4.0/−2.3	2.5/−5.7
Percent elongation		48.4/11.5	12.6/16.2	2.7/−6.4	10.6/6.9	−2.3/0.9
*Fatigue strength corresponding to*						
10^4^ cycles		10.5	−2.8	−6.8	−5.4	−6.8
10^5^ cycles		11.4	−4.7	−6.9	−6.8	−10.3
10^6^ cycles		11.8	−6.5	−7.0	−8.2	−13.7
*Mode I fracture toughness*		4.9	0.9	−2.4	−0.3	−0.6

^1^ The values shown before and after the slash symbol represent the synergistic indexes for the studied properties of the nanocomposites with total filler contents of 0.2 and 0.4 wt %, respectively.

**Table 3:** Parameters used in the modified Halpin-Tsai model.

MWCNTs					GNPs					Epoxy Matrix	
*l_CNT_* (nm)	*d_CNT_* (nm)	$\rho_{CNT}$ (g/cm^3^)	*E_CNT_* (MPa)		*l_GNP_* (nm)	*t_GNP_* (nm)	$\rho_{GNP}$ (g/cm^3^)	*E_GNP_* (TPa)		$\rho_{M}$ (g/cm^3^)	*E_M_* (MPa)
15,000	30	0.29	60,000		4000	5	1.39	1.0		1.14	3554

**Table 4:** Experimental results of fatigue tests performed on the study nanocomposites with various MWCNT/GNP ratios.

Filler Ratio (MWCNT:GNP)	Loading Level *r* (%)	Max. Applied Stress $\sigma_{\max}$ (MPa)	Fatigue Life *N_f_* (cycles)	Fatigue Life Curves			
				Fatigue Strength *a*	Fatigue Exponent *b*	Coef. of Determination *R*^2^	Pseudo Fatigue Limit, $\sigma_{e}$ (MPa)
0:00 (Neat Epoxy)	70	49.56	6,203	94.64	−0.0741	0.996	34
	65	46.02	17,995				
	60	42.48	50,281				
	55	38.94	134,088				
	50	35.4	639,238				
0:10	70	49.77	10,921	101.21	−0.0743	0.977	36.26
	65	46.22	54,603				
	60	42.66	109,242				
	55	39.11	432,690				
	50	35.55	>1,000,000				
1:9	70	57.12	7,741	107.98	−0.0697	0.989	41.22
	65	53.04	36,070				
	60	48.96	82,263				
	55	44.88	340,644				
	50	40.8	>1,000,000				
3:7	70	49.56	13,240	106.04	−0.0801	0.998	35.07
	65	46.02	37,232				
	60	42.48	84,852				
	55	38.94	254,360				
	50	35.4	924,930				
5:5	75	55.65	6,136	94.24	−0.0703	0.979	35.68
	70	51.94	25,820				
	65	48.23	79,835				
	60	44.52	145,844				
	55	40.81	581,860				
7:3	70	50.89	11,950	101.14	−0.0748	0.986	35.99
	65	47.26	21,436				
	60	44.82	86,999				
	55	39.99	189,423				
	50	36.35	>1,000,000				
9:1	70	49.7	14,842	109.37	−0.0834	0.995	34.55
	65	46.15	28,631				
	60	42.6	72,395				
	55	39.05	226,105				
	50	35.5	779,772				
10:0	75	56.85	6,700	100.57	−0.0659	0.978	40.46
	70	53.06	15,662				
	65	49.27	56,860				
	60	45.48	105,850				
	55	41.69	792,079				

**Table 5:** Experimental results of Mode I fracture toughness *K_IC_* for the studied nanocomposites with various MWCNT/GNP ratios. (MPa·$\sqrt{m}$).

MWCNT: GNP	0:0 (Neat Epoxy)	0:10	1:9	3:7	5:5	7:3	9:1	10:0
Fracture toughness ^1^ *K_IC_*	0.289 ± 0.018	0.861 ± 0.021 (+9.2)	0.904 ± 0.013 (+14.7)	0.871 ± 0.012 (+1.4)	0.843 ± 0.024 (+7.9)	0.863 ± 0.023 (+9.5)	0.862 ± 0.022 (+9.4)	0.868 ± 0.022 (+10.1)

^1^ The numbers shown in the brackets represent the percent improvement of the fracture toughness compared with the data of neat epoxy.

**Table 6:** Fitting results of the Paris law for the studied nanocomposites with various MWCNT/GNP ratios.

Filler Ratio MWCNT:GNP	0:0 (Neat Epoxy)	0:10	1:9	3:7	5:5	7:3	9:1	10:0
Coefficient, *C*	0.0299	0.0543	0.0809	0.1079	0.0316	0.0399	0.0605	0.1325
Exponent, *m*	3.432	4.732	5.956	5.450	4.789	4.202	4.691	6.189
Coef. of determination, *R^2^*	0.982	0.943	0.993	0.988	0.984	0.990	0.987	0.994

## References

[1] Ma P.C., Siddiqui N.A., Marom G., Kim J.K. (2010). Dispersion and functionalization of carbon nanotubes for polymer-based nanocomposites: A review. Compos. Part A-Appl. S. Manuf..

[2] Li B., Zhong W.H. (2011). Review on polymer/graphite nanoplatelet nanocomposites. J. Mater. Sci..

[3] Atif R., Shyha I., Inam F. (2016). Mechanical, thermal, and electrical properties of graphene-epoxy nanocomposites—A review. Polymers.

[4] Kumar A., Sharma K., Dixit A.R. (2019). A review of the mechanical and thermal properties of graphene and its hybrid polymer nanocomposites for structural applications. J. Mater. Sci..

[5] Shukla M.K., Sharma K. (2019). Effect of carbon nanofillers on the mechanical and interfacial properties of epoxy based nanocomposites: A review. Polym. Sci. Ser. B.

[6] Li J., Wong P.S., Kim J.K. (2008). Hybrid nanocomposites containing carbon nanotubes and graphite nanoplatelets. Mater. Sci. Eng. A.

[7] Kumar S., Sun L.L., Caceres S., Li B., Wood W., Perugini A., Maguire R.G., Zhong W.H. (2010). Dynamic synergy of graphitic nanoplatelets and multi-walled carbon nanotubes in polyetherimide nanocomposites. Nanotechnology.

[8] Yang S.Y., Lin W.N., Huang Y.L., Tien H.W., Wang J.Y., Ma C.C., Li S.M., Wang Y.S. (2011). Synergetic effects of graphene platelets and carbon nanotubes on the mechanical and thermal properties of epoxy composites. Carbon.

[9] Ren P.G., Di Y.Y., Zhang Q., Li L., Pang H., Li Z.M. (2012). Composites of ultrahigh-molecular-weight polyethylene with graphene sheets and/or MWCNTs with segregated network structure: Preparation and properties. Macromol. Mater. Eng..

[10] Chatterjee S., Nafezarefi F., Tai N., Schlagenhauf L., Nüesch F., Chu B. (2012). Size and synergy effects of nanofiller hybrids including graphene nanoplatelets and carbon nanotubes in mechanical properties of epoxy composites. Carbon.

[11] Das A., Kasaliwal G.R., Jurk R., Boldt R., Fischer D., Stöckelhuber K.W., Heinrich G. (2012). Rubber composites based on graphene nanoplatelets, expanded graphite, carbon nanotubes and their combination: A comparative study. Compos. Sci. Technol..

[12] Li W., Dichiara A., Bai J. (2013). Carbon nanotube–graphene nanoplatelet hybrids as high-performance multifunctional reinforcements in epoxy composites. Compos. Sci. Technol..

[13] Zhang S., Yin S., Rong C., Huo P., Jiang Z., Wang G. (2013). Synergistic effects of functionalized graphene and functionalized multi-walled carbon nanotubes on the electrical and mechanical properties of poly(ether sulfone) composites. Eur. Polym. J..

[14] Pradhan B., Srivastava S.K. (2014). Synergistic effect of three-dimensional multi-walled carbon nanotube–graphene nanofiller in enhancing the mechanical and thermal properties of high-performance silicone rubber. Polym. Int..

[15] Montes S., Carrasco P.M., Ruiz V., Cabañero G., Grande H.J., Labidi J., Odriozola I. (2015). Synergistic reinforcement of poly(vinyl alcohol) nanocomposites with cellulose nanocrystal-stabilized graphene. Compos. Sci. Technol..

[16] Li C., Li Y., She X., Vongsvivut J., Li J., She F., Gao W., Kong L. (2015). Reinforcement and deformation behaviors of polyvinyl alcohol/graphene/montmorillonite clay composites. Compos. Sci. Technol..

[17] Cui X., Ding P., Zhuang N., Shi L., Song N., Tang S. (2015). Thermal conductive and mechanical properties of polymeric composites based on solution-exfoliated boron nitride and graphene nanosheets: A morphology-promoted synergistic effect. ACS Appl. Mater. Interfaces.

[18] Al-Saleh M.H. (2015). Electrical and mechanical properties of graphene/carbon nanotube hybrid nanocomposites. Synth. Met..

[19] Moosa A.A., Sa A.R., Ibrahim M.N. (2016). Mechanical and electrical properties of graphene nanoplates and carbon nanotubes hybrid epoxy nanocomposites. Am. J. Mater. Sci..

[20] Wang F., Drzal L.T., Qin Y., Huang Z. (2016). Enhancement of fracture toughness, mechanical and thermal properties of rubber/epoxy composites by incorporation of graphene nanoplatelets. Compos. Part A-Appl. S. Manuf..

[21] Ghaleb Z., Mariatti M., Ariff Z. (2017). Synergy effects of graphene and multiwalled carbon nanotubes hybrid system on properties of epoxy nanocomposites. J. Reinf. Plast. Compos..

[22] Wang J., Jin X., Wu H., Guo S. (2017). Polyimide reinforced with hybrid graphene oxide@ carbon nanotube: Toward high strength, toughness, electrical conductivity. Carbon.

[23] Sahu S.K., Badgayan N.D., Samanta S., Sreekanth P.R. (2018). Quasistatic and dynamic nanomechanical properties of HDPE reinforced with 0/1/2 dimensional carbon nanofillers based hybrid nanocomposite using nanoindentation. Mater. Chem. Phys..

[24] Ribeiro H., Trigueiro J.P., Owuor P.S., Machado L.D., Woellner C.F., Pedrotti J.J., Jaques Y.M., Kosolwattana S., Chipara A., Silva W.M. (2018). Hybrid 2D nanostructures for mechanical reinforcement and thermal conductivity enhancement in polymer composites. Compos. Sci. Technol..

[25] Min C., Liu D., Shen C., Zhang Q., Song H., Li S., Shen X., Zhu M., Zhang K. (2018). Unique synergistic effects of graphene oxide and carbon nanotube hybrids on the tribological properties of polyimide nanocomposites. Tribol. Int..

[26] Shukla M.K., Sharma K. (2019). Effect of functionalized graphene/CNT ratio on the synergetic enhancement of mechanical and thermal properties of epoxy hybrid composite. Mater. Res. Express.

[27] Wang E., Dong Y., Islam M.Z., Yu L., Liu F., Chen S., Qi X., Zhu Y., Fu Y., Xu Z. (2019). Effect of graphene oxide-carbon nanotube hybrid filler on the mechanical property and thermal response speed of shape memory epoxy composites. Compos. Sci. Technol..

[28] Ren Y., Li F., Cheng H.M., Liao K. (2003). Tension–tension fatigue behavior of unidirectional single-walled carbon nanotube reinforced epoxy composite. Carbon.

[29] Zhang W., Picu R.C., Koratkar N. (2007). Suppression of fatigue crack growth in carbon nanotube composites. Appl. Phys. Lett..

[30] Yu N., Zhang Z.H., He S.Y. (2008). Fracture toughness and fatigue life of MWCNT/epoxy composites. Mater. Sci. Eng. A.

[31] Bortz D.R., Merino C., Martin-Gullon I. (2011). Carbon nanofibers enhance the fracture toughness and fatigue performance of a structural epoxy system. Compos. Sci. Technol..

[32] Loos M.R., Yang J., Feke D.L., Manas-Zloczower I. (2012). Enhanced fatigue life of carbon nanotube-reinforced epoxy composites. Polym. Eng. Sci..

[33] Rafiee M.A., Yavari F., Rafiee J., Koratkar N. (2011). Fullerene–epoxy nanocomposites-enhanced mechanical properties at low nanofiller loading. J. Nanopart. Res..

[34] Rafiee M.A., Rafiee J., Srivastava I., Wang Z., Song H., Yu Z.Z., Koratkar N. (2010). Fracture and fatigue in graphene nanocomposites. Small.

[35] Ladani R.B., Bhasin M., Wu S., Ravindran A.R., Ghorbani K., Zhang J., Kinloch A.J., Mouritz A.P., Wang C.H. (2018). Fracture and fatigue behaviour of epoxy nanocomposites containing 1-D and 2-D nanoscale carbon fillers. Eng. Fract. Mech..

[36] Ismail H., Ramly A.F., Othman N. (2011). The effect of carbon black/multiwall carbon nanotube hybrid fillers on the properties of natural rubber nanocomposites. Polym.-Plast. Technol..

[37] Dong B., Liu C., Lu Y., Wu Y. (2015). Synergistic effects of carbon nanotubes and carbon black on the fracture and fatigue resistance of natural rubber composites. J. Appl. Polym. Sci..

[38] Shokrieh M.M., Esmkhani M., Haghighatkhah A.R., Zhao Z. (2014). Flexural fatigue behavior of synthesized graphene/carbon-nanofiber/epoxy hybrid nanocomposites. Mater. Design.

[39] ASTM International (2014). Standard Test. Method for Tensile Properties of Plastics.

[40] ASTM International (2014). Standard Test. Methods for Plane-Strain Fracture Toughness and Strain Energy Release Rate of Plastic Materials.

[41] ASTM International (2015). Standard Test. Method for Measurement of Fatigue Crack Growth Rates.

[42] Zhang X., Liu T., Sreekumar T.V., Kumar S., Moore V.C., Hauge R.H., Smalley R.E. (2003). Poly(vinylalcohol)/SWNT Composite Film. Nano Lett..

[43] Zhao X., Zhang Q., Chen D. (2010). Enhanced mechanical properties of graphene-based poly(vinyl alcohol) composites. Macromolecules.

[44] Bannantine J.A., Comer J.J., Handrock J.L. (1990). Fundamentals of Metal Fatigue Analysis.

[45] Szeluga U., Kumanek B., Trzebicka B. (2015). Synergy in hybrid polymer/nanocarbon composites. A review. Compos. Part A-Appl. S. Manuf..

[46] Bortz D.R., Heras E.G., Martin-Gullon I. (2013). Impressive Fatigue Life and Fracture Toughness Improvements in Graphene Oxide/Epoxy Composites. Macromolecules.

[47] Chandrasekaran S., Sato N., Tölle F., Mülhaupt R., Fiedler B., Schulte K. (2014). Fracture toughness and failure mechanism of graphene based epoxy composites. Compos. Sci. Technol..

[48] Park Y.T., Qian Y., Chan C., Suh T., Nejhad M.G., Macosko C.W., Stein A. (2015). Epoxy toughening with low graphene loading. Adv. Funct. Mater..

[49] Cha J., Kim J., Ryu S., Hong S.H. (2019). Comparison to mechanical properties of epoxy nanocomposites reinforced by functionalized carbon nanotubes and graphene nanoplatelets. Compos. Part. B-Eng..

[50] Kumar A., Li S., Roy S., King J.A., Odegard G.M. (2015). Fracture properties of nanographene reinforced EPON 862 thermoset polymer system. Compos. Sci. Technol..

[51] Moosa A.A., Kubba F., Raad M., Ramazani A. (2016). Mechanical and thermal properties of graphene nanoplates and functionalized carbon-nanotubes hybrid epoxy nanocomposites. Am. J. Mater. Sci..

